# Development of a toolkit for *piggyBac*-mediated integrative transfection of the human filarial parasite *Brugia malayi*

**DOI:** 10.1371/journal.pntd.0006509

**Published:** 2018-05-21

**Authors:** Canhui Liu, Amruta S. Mhashilkar, Johan Chabanon, Shulin Xu, Sara Lustigman, John H. Adams, Thomas R. Unnasch

**Affiliations:** 1 Center for Global Health Infectious Disease Research, Department of Global Health, University of South Florida, Tampa, FL, United States of America; 2 Molecular Parasitology, New York Blood Center, New York, NY, United States of America; McGill University, CANADA

## Abstract

**Background:**

The human filarial parasites cause diseases that are among the most important causes of morbidity in the developing world. The elimination programs targeting these infections rely on a limited number of drugs, making the identification of new chemotherapeutic agents a high priority. The study of these parasites has lagged due to the lack of reverse genetic methods.

**Methodology/Principal findings:**

We report a novel co-culture method that results in developmentally competent infective larvae of one of the human filarial parasites (*Brugia malayi*) and describe a method to efficiently transfect the larval stages of this parasite. We describe the production of constructs that result in integrative transfection using the *piggyBac* transposon system, and a selectable marker that can be used to identify transgenic parasites. We describe the production and use of dual reporter plasmids containing both a secreted luciferase selectable marker and fluorescent protein reporters that will be useful to study temporal and spatial patterns of gene expression.

**Conclusions/Significance:**

The methods and constructs reported here will permit the efficient production of integrated transgenic filarial parasite lines, allowing reverse genetic technologies to be applied to all life cycle stages of the parasite.

## Author summary

The human filarial parasites are the causative agents of lymphatic filariasis (elephantiasis) and onchocerciasis (river blindness) that are some of the most important causes of morbidity worldwide. Both of these diseases have been targeted for elimination by the international community. However, the elimination programs for both diseases rely on long-term community-wide distribution of a limited number of drugs, which is logistically difficult to achieve, and vulnerable to failure if resistance to the current drugs develops. Thus, additional research on the biology of these parasites is urgently needed. A large obstacle to research on these organisms has been the inability to employ reverse genetic methods and to develop integrated transgenic parasite lines. In this manuscript, we report the development of a novel co-culture method and constructs that overcome this hurdle. Together these can be used to efficiently produce integrated transgenic parasites.

## Introduction

The human filarial parasites cause two of the most important neglected tropical diseases of mankind, lymphatic filariasis (elephantiasis) and onchocerciasis (river blindness). Both have been identified as diseases that may be eliminated as a public health problem [1], and both are targets of elimination campaigns that rely primarily or exclusively on mass drug treatment of the afflicted populations. Despite the success of these programs, there remains a pressing need for new chemotherapeutic agents and alternative modalities to treat these infections, as most programs rely on a limited number of drugs that must be given repeatedly over the course of many years, leading to the potential for resistance development [2].

The study of many pathogens has benefitted enormously from reverse genetic technologies. Unfortunately, the human filarial parasites have lagged behind most other organisms in these technologies, for several reasons. The human filaria are all obligate parasites; none of their life cycle stages can be cultured outside of the host. Only *Brugia malayi* (a parasite causing human filariasis in Southeast Asia) can be maintained throughout the full mammalian life cycle in an animal host, most efficiently in the jird (the gerbil *Meriones unguiculatus*) [3–6]. The difficult biology of *B*. *malayi* has made development of a stable transfection system an elusive goal. Mechanical transfection methods result in damage to the parasite cuticle, which causes the death of the parasite when it is re-introduced into its host. The mature intact cuticle is also impermeable to chemical transfection reagents, pantropic retroviruses and lentiviruses. Nonetheless, *B*. *malayi* larvae were found susceptible to CaCl_2_ transfection during the L3-L4 molt in culture [7]. Unfortunately, the resulting L4 larvae were not developmentally competent when introduced into jirds [7]. Co-injection of CaCl_2_ co-precipitates and L3 parasites into the peritoneal cavity of a naïve jird did result in developmentally competent transfected parasites [7], but this process was very inefficient, and by analogy to what has been found in other nematodes, the transgenes in these parasites were likely maintained in extrachromosomal arrays [8]. Expression of genes maintained in this manner are often silenced after the F1 generation [8]. To obtain stable transgenic lines where expression of transgenes is reliably maintained, genomic integration is necessary [8].

One of the most successful approaches used to obtain integrative transgenesis has been heterologous transposon-based systems, especially the *piggyBac* transposase-transposon system [9–12]. In this system, two plasmids are used. One, the helper plasmid, encodes the transposase that mobilizes the transposon and the second consists of the *piggyBac* transposon inverted terminal repeats (ITRs) flanking a selectable marker and the gene under study [13]. Co-transfection with both plasmids results in expression of the transposase, which mobilizes the ITRs in the second plasmid, resulting in insertion of the ITR cassette into the genome at sites containing the sequence TTAA [13]. The helper plasmid is not incorporated into the genome and is lost during cell replication, resulting in stable transgenic lines. Here we describe a culture method for efficient production of developmentally-competent transgenic parasites. We describe the adaptation of the *piggyBac* system to *B*. *malayi*, which results in the integration of transgenes into the *B*. *malayi* genome. We identify a selectable marker that can be used to identify transfected parasites and describe the production of constructs that contain both a selectable marker and fluorescent reporter proteins that can be used to study protein targeting and spatial expression. Together, these advances represent tools that will permit investigators to "knock in" genes in all life cycle stages the human filaria.

## Materials and methods

### Plasmid constructs

**Fig 1:**
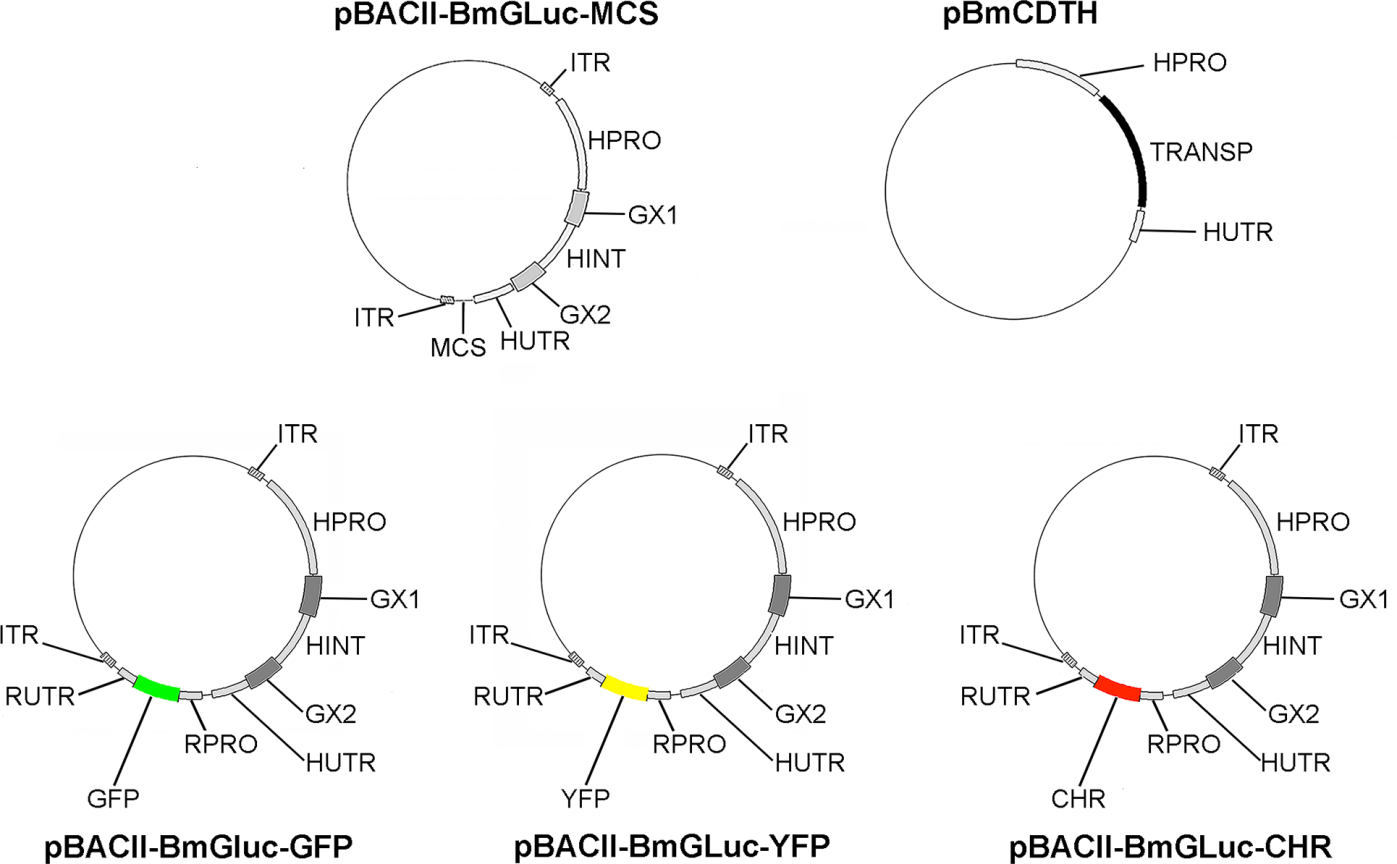
Maps of plasmid constructs used in this study. ITR = inverted terminal repeat; HPRO = *BmHSP70* promoter; GX1 = *Gaussia* luciferase ORF exon 1; HINT = *BmHSP70* intron 1; GX2 = *Gaussia* luciferase ORF exon 2; HUTR = *BmHSP70 3' UTR;* MCS = multiple cloning site (*Bcl* 1—*Bgl* II—*Mlu* 1—*Cla* 1—*Sma* 1—*Aat* II—*Nde* I—*Bcl* II); TRASNP = *piggyBac* transposase ORF; RPRO = *Bmrps12* promoter; RUTR = *Bmrps12* 3' UTR; GFP = Green Fluorescent Protein ORF; YFP = Yellow Fluorescent Protein ORF; CHR = Cherry Red ORF.

The *B*. *malayi piggyBac* system was based upon two plasmids: pBACII-BmGluc-MCS and pBmCDTH. The structure of these plasmids is given in Fig 1. pBACII-BmGluc-MCS contains a *Gaussia princeps* secreted luciferase (*GLuc*) selectable marker whose expression is controlled by the *Brugia malayi* HSP70 (*Bmhsp70*) promoter and *Bmhsp70* 3’ UTR as well as a multiple cloning site, both of which are flanked by the *piggyBac* inverted terminal repeats (ITRs). pBmCDTH contains the *piggyBac* transposase under the control of the *Bmhsp70* promoter.

The construction of pBACII-BmGluc-MCS is summarized in S1 Fig. Two plasmids were used in the construction of pBACII-BmGluc-MCS; pL-BACII-EDGH and pBmHS70GLuc. The construction and verification of pBmHSP70GLuc (GenBank Accession # HQ388295) was described previously [7]. The expression cassette in pBmHSP70GLuc, consisting of the *Bmhsp70* promoter, the GLuc ORF and the *Bmhsp70* 3’UTR was amplified with primers containing synthetic *Avr* II restriction sites. Similarly, the sequence between positions 3950 and 985 of pL BACII-EGDH, representing the plasmid backbone and ITRs, was amplified with primers containing synthetic *Avr* II restriction sites. The sequence of the primers used for these reactions are provided in Table 1.

**Table 1:** Primers used in the production of the *piggyBac* plasmids.

Primer	Sequence*	Purpose
HSP70/Gluc F	5' GGCCTAGGCTGAAGGGCAATACCACTAC 3'	Amplification of *Bmhsp70* GLuc cassette
HSP70/Gluc R	5' GGCCTAGGCTAGAACAATATTCACAAGGAC 3'	
BACllF	5' TTTTGGCCTAGGGTTATCACGTAAGTAGAACAT 3'	Amplification of pL BACII backbone
BACllR	5' TTTTGGCCTAGGGATAAAAGTTTTGTTACTTTATAGAAG 3'	
Bcl1 F	5’ ACGCGGTCGTTATAGTTCATGATCAGTGACACTTACCGCA 3’	Introduction of a *Bcl* 1 site into pBACII-BmGLuc
Bcl1 R	5’ TGCGGTAAGTGTCACTGATCATGAACTATAACGACCGCGT 3’	
MCS-F	5' GATCACATATGGACGTCCCCGGGATCGATACGCGTAGATCT 3'	MCS oligonucleotides
MCS R	5' GATCAAGATCTACGCGTATCGATCCCGGGGACGTCCATATG 3'	
BmRPS12-PRO F	5’ GGGCATATGGCTTAAGGAGAATTTTTAAAAAACTATAGAG 3’	Amplification of *Bmrps12* promoter
BmRPS12-PRO R	5’ GGGCATATGCATGTTCAGCAGTTGTCCTCGAT 3’	
BmRPS12-UTR F	5’ GGGAGATCTTGACTATTGTTTGTTTATTGTTTGTATTGAAG 3’	Amplification of *Bmrps12* 3' UTR
BmRPS12-UTR R	5’ GGGAGATCTGCCCAAGCAATTTCGAATGA 3’	
CHR-YFP F	5’ GGGACGCGTGTGCATTTAGGACATCTCAGTC 3’	Amplification of YFP & CHR ORFs
CHR-YFP F	5’ GGGACGCGTCTTGTACAGCTCGTCCATGC 3’	
GFP F	5’ GGGACGCGTCGGAGCAAGCTTGATTTAGG 3’	Amplification of GFP ORF
GFP R	5’ GGGACGCGTTGGTTTGTCCAAACTCATCAA 3’	
Trans F	5’ GGTTAATTAAATGGGTAGTTCTTTAGACGAT 3’	Amplification of transposase ORF
Trans R	5’ GGTTAATTAAATGGGTAGTTCTTTAGACGAT 3’	
BmGluc1701 F	5’ TTTTTGGTTAATTAAGCGGCCGCTCGAGCATGCAT 3’	Deletion of GLuc ORF
BmGluc688 R	5’ TTTTTGGTTAATTAAATTTGCTGTATTTCGAGAAACG 3’	

* underlined sequences indicate restriction sites introduced to facilitate subsequent cloning.

The PCR amplification reactions utilized *Pfu* Ultra II Fusion HS DNA Polymerase (Agilent, Santa Clara, California). The amplification reactions contained 1 μl of *Pfu* polymerase and 5 ng of template plasmid DNA in a solution containing 1x *Pfu* Ultra II Fusion HS buffer (provided by the manufacturer), 0.2 μM of each primer and 250 μM of each dNTP. Cycling conditions consisted of an initial denaturation step of 2 minutes at 94°C, followed by 30 cycles consisting of 30 sec at 94°C, 30 sec at 55°C and 3 min at 72°C. The resulting products were digested with *Avr* II and ligated to produce the parental plasmid. This plasmid (pBACII-BmGluc) was then mutated to insert a *Bcl* I restriction site between the end of the HSP70 3’UTR and the ITR, using the GENEART Site-Directed Mutagenesis System kit (Thermo Fisher) following the manufacturer’s instructions. The primers used to introduce this mutation are given in Table 1. Individual clones from the mutagenesis reaction were isolated and the mutation confirmed by DNA sequence analysis. The mutated plasmid was digested with *Bcl* I and ligated with a double-stranded oligonucleotide encoding a multiple cloning site with *Bcl* 1 overhangs, yielding pBACII-BmGluc-MCS. The sequences of these oligonucleotides are provided in Table 1. The sequence of the resulting plasmid was confirmed, and the functionality of the GLuc marker further confirmed by biolistic transfection of *B*. *malayi* embryos [14].

The strategy used to produce pBmCDTH is summarized in S2 Fig. Construction of pBmCDTH began with pCDTH (a plasmid containing the *piggyBac* transposase ORF) and pBmHSP70GLuc. The transposase ORF was amplified from pCDTH using primers containing synthetic *Pac* 1 restriction sites. Similarly, the plasmid backbone of pBmHSP70GLuc was amplified, deleting the GLuc ORF from the construct. The primers used contained synthetic *Pac* 1 sites at their 5' ends. The sequences of the primers used are given in Table 1; amplification conditions were identical to those used to amplify the expression cassette in pBmHSP70GLuc. Following digestion with *Pac* 1, the transposase ORF was ligated to the plasmid backbone, resulting in the transposase ORF flanked by the *Bmhsp70* promoter and the *Bmhsp70* 3’ UTR. The DNA sequence of the final plasmid (pBmCDTH) was confirmed.

pBACII-BmGluc-MCS was used to as a backbone to produce a series of dual reporter plasmids expressing both the *Gluc* selectable marker and different fluorescent protein tags. The structure of these plasmids is given in Fig 1, and their construction is summarized in S3 Fig. To construct these plasmids, the *Bmrps12* promoter [15] was amplified from *B*. *malayi* genomic DNA using primers with synthetic *Nde* 1 restriction sites on their 5' ends. Similarly, the *Bmrps12* 3'UTR was amplified using primers with synthetic *Bgl* II sites on their ends. The sequences of the primers are provided in Table 1. Amplification reactions were carried out as described above, using 100 ng of *B*. *malayi* genomic DNA as a template. Cycling conditions consisted of an initial denaturation step at 94°C for 3 min, followed by 30 cycles consisting of 94°C for 30 sec, 55°C for 40 sec and 68°C for 90 sec. The reaction was completed with an extension of 10 min at 68°C. The amplicons were cloned, and their DNA sequence confirmed. They were then cloned into the *Nde* 1 and *Bgl* II sites of the pBACII-BmGLuc MCS. Finally, the Green Fluorescent Protein (GFP), Yellow Fluorescent Protein (YFP) and Cherry Red (CHR) ORFs were amplified from plasmids obtained from Addgene (Cambridge, MA) with primers containing synthetic *Mlu* 1 sites at their 5' ends. The sequences of the primers are provided in Table 1. The amplicons were cloned, their sequence confirmed, and the ORFs then subcloned into the *Mlu* 1 site of the pBACII-BmGLuc MCS. The sequence of all three plasmids was then confirmed and the functionality of the GLuc markers further confirmed by biolistic transfection of *B*. *malayi* embryos [14].

### Lipofection and implantation of *B*. *malayi* infective larvae (L3)

*B*. *malayi* infective larvae (L3) were obtained from the Filarial Research Reagent Repository (FR3). Prior to the arrival of the L3, the individual wells of a 24-well tissue culture plate were seeded with 1x10^5^ Bovine Embryo Skeletal Muscle (BESM) cells/well. The cells were cultured for 1–2 days in Minimal Essential Media (MEM) containing 20% fetal bovine serum (FBS) until they reached 70–90% confluency. Upon receipt, the L3 were washed five times with a solution consisting of RPMI 1640 medium containing 0.1x Antibiotic Antimycotic solution (Gibco) 10 μg/ml gentamycin and 2 μg/ml Ciprofloxin. The L3 (500 maximum) were then dispersed in 5 ml of RPMI 1640 media containing 25 mM HEPES, 20% fetal calf serum, 20 mM glucose, 24 mM sodium bicarbonate, 2.5 μg/ml amphotericin B, 100 U/ml penicillin, 100 U/ml streptomycin, 40 μg/ml gentamicin, 2 μg/ml Ciprofloxin and 2 μg/ml Fortaz (CF-RPMI). The L3 were allowed to settle and all but 1ml of media removed. The media on the feeder cells was replaced with CF-RPMI and transwells (Costar, 3.0 um pore size) were placed in the wells. The dispersed L3 were then aliquoted among the transwells so that each transwell contained approximately 100 L3. Additional CF-RPMI was added to the transwell to bring the total volume in the transwell to 500 μl.

Transfections were initiated by diluting 24 μl Lipofectamine LTX reagent (Thermo Fisher) in 300 μl Opti-MEM medium (Thermo Fisher). A total of 3 μg of the *piggyBac* ITR plasmid DNA and 3 μg pBmCDTH plasmid DNA were then added to 300 μl Opti-MEM medium; 6 μl PLUS Reagent was then added to the DNA solution. The diluted DNA was then combined with 300 μl of the diluted Lipofectamine LTX Reagent to prepare the DNA micelles. The mixture was incubated for 5 minutes at room temperature. A total of 50 μl of the micelle solution was then added to each well containing the L3. The L3 were then cultured at 37°C under 5% CO_2_. The feeder cell medium was changed and an additional 50 μl of freshly prepared micelle solution was added to the transwells on a daily basis. On day 5, molting of the L3 was induced by the inclusion of ascorbic acid to a final concentration of 75 μM in the feeder cell medium. The L3 were incubated for a total of eight days. In the initial experiments to assess the efficiency of the larval transfection over the time in culture, control cultures included cells cultured in the absence of L3 and L3 cultured in the absence of cells. In the initial experiments, three biological replicates were examined for each treatment (larvae alone, cells alone and larvae plus cells).

To assess the developmental competence of the co-cultured larvae, 8 day transfected larvae prepared as described above (3 days following induction of molting when secreted GLuc activity was maximal) were collected and injected intraperitoneally into two naive jirds, using an 18 gauge needle. Approximately 100 L3 were injected into each animal. The parasites were allowed to develop for 110 days and the animals examined for the production of microfilariae once every five days for 20 days by peritoneal lavage. The animals were then euthanized and their peritoneal cavity examined for adult stage parasites. Recovered microfilariae were cultured individually in 50 μl of CF-RPMI for 48 hours in 384 well plates and the medium assayed for secreted luciferase activity as described below. A detailed protocol describing the lipofection procedure has been deposited with www.protocols.io, at https://protocols.io/view/brugia-malay-transfection-nrtdd6n.

### Analysis of reporter expression in transfected parasites

Transfected parasites were analyzed for expression of the secreted luciferase and expression of the fluorescent proteins. To analyze for secreted luciferase, 20 μl of culture medium (from both the L3 cultured parasites and the individual microfilaria cultured in the 384 well plates) was removed from the wells containing the cultured parasites. The medium was mixed with 100 μl of LARII and 100 μl of Stop & Glow buffer (Promega) in a disposable glass test tube. A total of 2 μl of Dual-Glo Stop & Glo reagent was added to the tube and the mixture was mixed by flicking the tube. The mixture was incubated for 10 minutes and emitted light read in a luminometer.

Roughly five day 8 parasites transfected with the dual reporter plasmids (3 days following induction of molting when secreted GLuc activity was maximal) in 20–30 μl of medium were mounted on a glass slide, covered by a coverslip, and viewed on inverted Zeiss Observer fluorescent automated microscope equipped with 100x objective. To detect GFP fluorescence in the worms, the sample was excited with 395nm wavelength light (emission peak at 509nm); YFP fluorescence was excited with 514nm wavelength light (emission peak at 527nm), and CHR signal was detected after sample excitation with 558nm wavelength light (emission peak at 583nm). Images were collected and processed using AxioVision v4.8 software. A total of 25 parasites transfected with each construct (i.e. 5 slides) were examined for each reporter plasmid transfection.

### PCR amplification of *piggyBac* insertion sites from individual microfilariae

Individual microfilariae were placed in 100 μl of nuclease-free deionized water and subjected to two freeze-thaw cycles. The samples were then heated to 100°C for 10 minutes and 1μl of proteinase K (1mg/ml) was added. The samples were incubated at 55°C for 50 minutes and the proteinase K inactivated by heating the samples to 95°C for 10 minutes.

Insertion sites were amplified from the DNA isolated from the individual parasites using a Thermal Asymmetric Interlaced (TAIL) PCR protocol. This process utilized four independent reactions, each of which contained a different random sequence amplification primer and a primer derived from the sequence located internally to the ITR2 of the *piggyBac* insertion cassette. The sequences of the primers used in these reactions are given in Table 2. The first amplification reactions contained 5 μl of the isolated DNA solution, 0.2 μM *piggyBac* 1548 primer and each of the individual random amplification primers at a concentration of 2 μM, in a total volume of 25 μl. Amplification reactions were prepared using GoTaq DNA polymerase and buffers (Promega) following the manufacturer's instructions. The reaction began with an incubation at 95°C for 5 min, followed by 5 cycles consisting of 94°C for 30 sec, 65°C for 1 min and 72°C for 2 min. This was followed by 1 cycle of 94°C for 30 sec, 25°C for 2 min, ramping to 72°C over 2 min and then 72°C for 2 min. This was followed by 15 cycles of nine steps: 94°C for 30 sec, 65°C for 1 min, 72°C for 2 min, 94°C for 30 sec, 65°C for 1 min, 72°C for 2 min, 94°C for 30 sec, 44°C for 1 min and 72°C for 2 min. The reaction was completed with a final extension for 5 min at 72°C. The resulting product from each of the four first reactions was then diluted 1/40 in PCR grade water and 2 μl was used as a template in four 25 μl nested reactions using the GoTaq DNA polymerase and buffers as described above. The nested reactioncontained 0.2 μM of the *piggyBac* 1547 primer and 2 μM of the same AD primer employed in the initial reaction. Cycling conditions consisted of 15 cycles each containing the following 9 steps: 94°C for 30 sec, 65°C for 1 min, 72°C for 2 min, 94°C for 30 sec, 65°C for 1 min, 72°C for 2 min, 94°C for 30 sec, 45°C for 1 min, 72°C for 2 min. The reaction was completed with a final extension of 72°C for 5 min. The product from this reaction was diluted 1/10 in PCR grade water and 2 μl was used as a template in a second set of nested amplification reactions, again using the GoTaq DNA polymerase and buffers, in a final volume of 25 μl. This reaction contained 0.2 μM of *piggyBac* 1558 primer and 2 μM of the same AD primer employed in the initial reaction. Cycling conditions consisted of 40 cycles consisting of 94°C for 30 sec, 65°C for 1 min and 72°C for 2 min. The reaction was completed with a final extension of 72°C for 5 min. The products from the third reaction were analyzed by electrophoresis on a 1.5% agarose gel. Bands from reactions producing distinct bands were cut out and purified. The DNA sequence of the resulting purified amplicons was then determined by a commercial sequencing service using the *piggyBac* 1558 primer. The resulting sequences were compared to the *B*. *malayi* complete genome sequence in Wormbase [16] to identify the location of the insertion sites.

**Table 2:** Primers used in the TAIL PCR.

Primer	Sequence	Purpose
*piggyBac* 1548	5' CATTGACAAGCACGCCTCAC 3'	Primary ITR2 specific primer
*piggyBac* 1547	5' CTCCAAGCGGCGACTGAG 3'	Secondary ITR2 specific primer
*piggyBac* 1558	5' AGATGTCCTAAATGCACAGCGAC 3'	Tertiary ITR2 specific primer
AD 1	5' NTCGA(G/C)T(A/T)T(G/C)G(A/T)GTT 3'	Degenerate primer 1
AD 2	5' NGTCGA(G/C)(A/T)GANA(A/T)GAA 3'	Degenerate primer 2
AD 3	5' (A/T)GTGNAG(A/T)ANCANAGA 3'	Degenerate primer 3
AD 4	5' AG(A/T)GNAG(A/T)ANCA(A/T)AGG 3'	Degenerate primer 4

### Ethics statement

The protocols involving animals were reviewed by the Institutional Animal Care and Use Committee of the University of South Florida and were approved under protocol # R IS00003568. The University of South Florida’s animal research program is consistent with the recommendations of the Guide for the Care and Use of Laboratory Animals, Animal Welfare Act, Public Health Service Policy on Humane Care and Use of Laboratory Animals and the IACUC Principles and Procedures. The University of South Florida’s OLAW assurance number is D16-00589(a4100-01) and its USDA registration is 58-R-0015.

## Results

**Fig 2:**
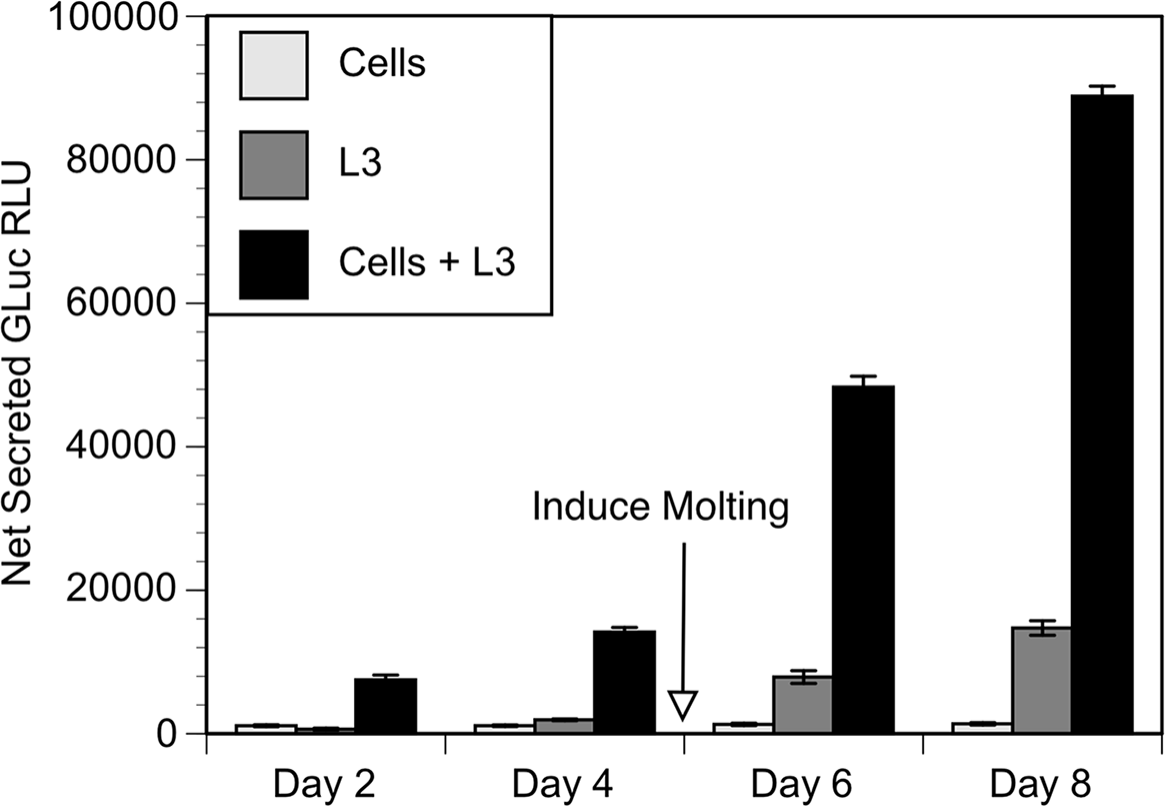
Secreted GLuc activity in cultured transfected L3. L3 = cultured in absence of cells. Cells = BESM cells alone. L3 + cells = L3 cultured and transfected over BESM monolayer. Columns represent mean activity and error bars standard deviation of data obtained from three biological replicates (i.e. independent transfections carried out individual transwells) per treatment. The transfections employing L3 alone and L3 + cells contained approximately 100 larvae per transwell. Values represent net counts calculated by subtracting the relative light units (RLU) seen in media collected from untransfected cells, L3 and cells + L3 respectively.

In previous work, it was demonstrated that L3 could be transfected in culture, and that the process was most efficient when the L3 were induced to molt by the addition of ascorbic acid to the cultures [7]. However, the resulting *in vitro* induced molted larvae were developmentally incompetent [7]. Recently, a report suggested that co-culture of *Onchocerca volvulus* L3 larvae in the presence of Bovine Embryo Skeletal Muscle (BESM) cells resulted in enhanced molting and continued development of the resulting L4 parasites towards adults [17]. We hypothesized that co-culture might also enhance the efficiency of transfection and survival of cultured *B*. *malayi* larvae. We co-cultured *B*. *malayi* L3 in transwells over a monoloyer of BESM cells and then exposed them to micelles prepared with a commercial lipofection reagent (lipofectamine). The micelles were loaded with the plasmid pBmHSP70GLuc [7], which consists of a secreted Gaussia luciferase (*GLuc*) ORF flanked by the *B*. *malayi hsp* 70 promoter and 3'UTR. The first intron of the *B*. *malayi hsp* 70 gene was also inserted into the *GLuc* ORF. The intron contains the sequences necessary and sufficient to direct trans-splicing in *B*. *malayi* [18] ensuring that the nascent mRNA derived from the *GLuc* ORF was properly processed. Molting was induced at day 5. Controls for this experiment included transfections carried out on the cells cultured alone and L3 cultured in the absence of cells. The amount of GLuc activity secreted into the medium was significantly higher in L3 cultured in the presence of cells than in L3 cultured in the absence of cells, or the cells cultured alone at all time points assayed during the experiment (p < 0.001; t test; Fig 2). Net secreted GLuc activity in parasites cultured in the presence of the BESM cells at day eight averaged 7x greater than the activity observed in parasites transfected and cultured in the absence of the cells, and 28x greater than in the cells cultured alone (Fig 2). This suggested that L3 cultured in the presence of the BESM cells were either more efficiently transfected or produced more secreted luciferase upon becoming transfected than those cultured in the cell's absence.

To determine if the L4 produced by co-culturing L3 in the presence of BESM cells were developmentally competent, parasites were transfected as described above, induced to molt on day 5 and after 8 days in culture were injected into the peritoneum of two naïve jirds. The parasites were allowed to develop for 130 days and the parasites recovered. A total of 9 adult parasites (males and females) were recovered from one of the infected animals, while 21 adult parasites were recovered from the second animal. This recovery rate is similar to that observed in gerbils intraperitoneally infected with freshly prepared uncultured L3 (18%) [19]. Microfilariae were also observed in the peritoneal cavity of the animals infected with the transfected larvae, suggesting the adult females were fertile. These data suggested that the lipofection-treated parasites produced in co-culture with BESM cells were developmentally competent.

**Fig 3:**
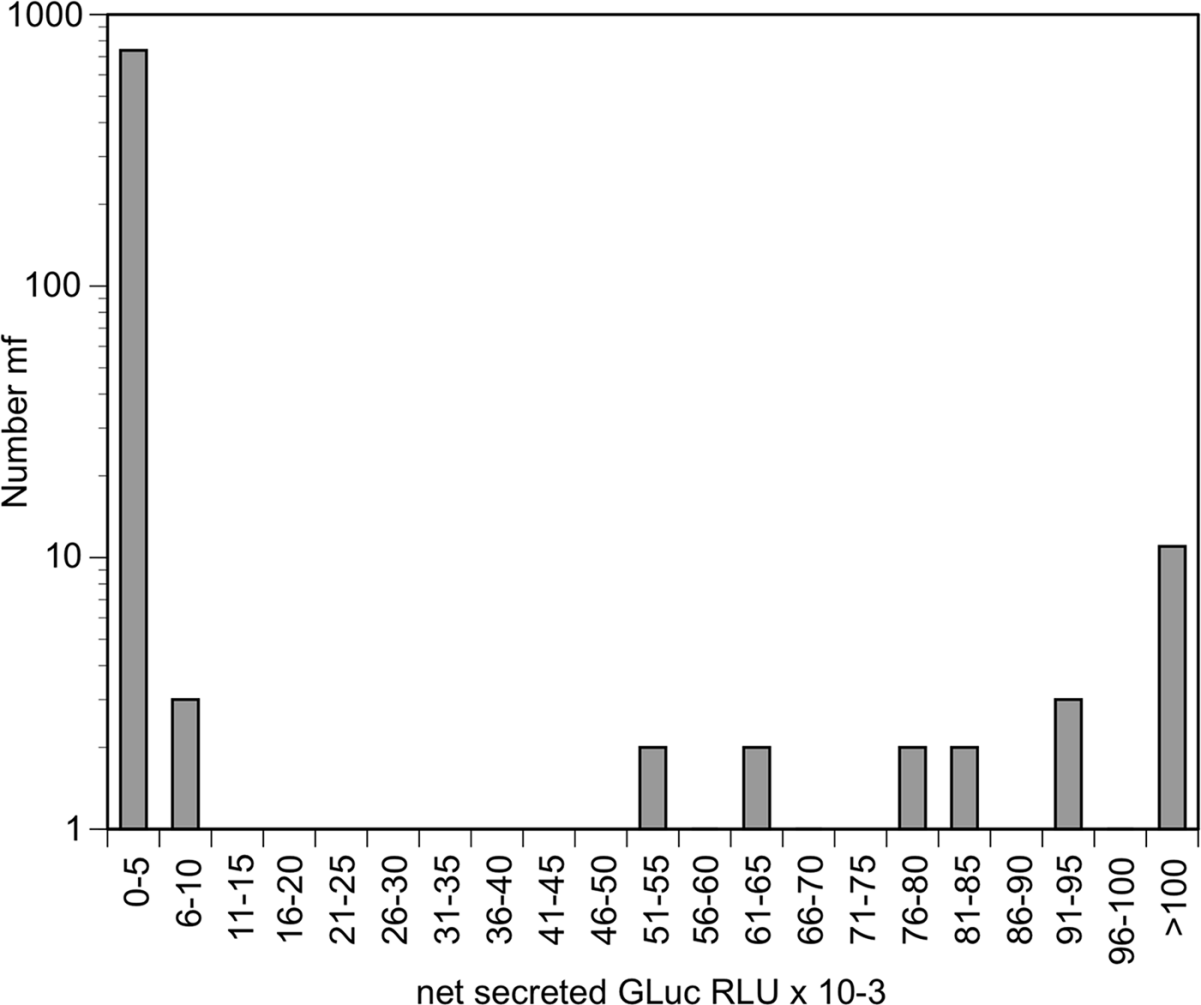
Secreted GLuc activity in cultures of individual microfilaria derived from adult parasites transfected in the L3-L4 stage. Bars represent the number of individual microfilariae producing secreted GLuc activity in the range shown. Note that the y-axis is logarithmic.

To determine if: 1. it was possible to obtain integrative transfection using the *piggyBac* system in co-cultured parasites; 2. If transgenesis was maintained in the F1 generation and; 3. if GLuc could be used as a selectable marker to identify transgenic parasites, the HGPRT and GFP genes in the *piggyBac* ITR vector pL-BACII-EDGH were replaced with the pBmHSP70/Gluc reporter cassette. A construct placing the ORF of the *piggyBac* transposase under the control of the *B*. *malayi* HSP70 promoter was also prepared. *B*. *malayi* larvae were lipofected with both plasmids and molting was induced by the addition of ascorbic acid on day 5, as described above. The parasites were cultured until day 8 and approximately 100 of these parasites were injected interperitoneally into each of two naïve jirds. Microfilariae recovered from the peritoneal cavities were manually individually isolated. Each microfialria was placed into a well of a 384 well plate (two plates total, with each plate containing 384 individual microfilaria from a one infected animal) and assayed for the presence of secreted GLuc activity after 24 hours in culture. The net number of RLUs seen in the medium of the cultured individual microfilaria exhibited a distinct bimodal distribution (Fig 3). The means of the secreted GLuc activity in these two groups were significantly different from one another (p = 0.011; t test).

Most of the individual microfilaria (741/766; 97%) produced GLuc activity that was near background levels (Fig 3). However, some of the individual microfilaria secreted relatively large amounts of GLuc into the medium. A total of 11/384 microfilariae collected from one animal produced secreted GLuc activity that was at least ten times over what was seen in the wells containing microfilaria producing near background levels of secreted Gluc activity, while 14/384 microfilaria from the second animal did so (p > 0.05; χ^2^ test). A total 11 of the individual microfilaria from both animals produced levels of secreted luciferase activity that exceeded 100,000 RLU (Fig 3).

DNA from eight microfilariae that produced >100,000 RLU of secreted GLuc activity were then used as a template in an asymmetric interlaced PCR to determine if the parasites contained the transgene, if it had been integrated into the genome and if so where this had occurred. A single distinct band was obtained from all eight of the interlaced reactions. The DNA sequence of these amplicons was the determined. All 8 contained the expected ITR sequences of the *piggyBac* transposon flanked by the expected TTAA integration site. The size of the amplicons obtained from the PCR amplification reactions, the percentage match of these amplicons to their putative integration sites (trimmed of the transposon sequences) and the location of the insertions are provided in Table 3. The integration sites were all located in intergenic regions on the autosomes of the *B*. *malayi* genome. The insertion sites present in all but microfilaria 6 and 7 appeared to be unique in the genome, as no significant matches were found to the amplicon sequences apart from the ones given in Table 3. Highly significant matches were seen to the 5s rRNA spacer sequence in the genome for microfilaria 6 and 7, suggesting they inserted into this repeated sequence element. Thus, the exact location of the insertion points for these microfilaria could not be determined.

**Table 3:** Location of *piggyBac* insertions in genomes of individual microfilaria.

Mf	Approximate Fragment size (bp)	Match/ Sequenced (%)	Chromosome	location
1	320	215/218 (99%)	2	between Bm8650 and Bm1146
2	420	321/321 (100%	3	between Bm17591 and Bm12776
3*	350	255/255 (100%)	1	between Bm10776 and Bm8585
4	350	249/249 (100%)	2	between Bm4108 and Bm8650
5*	350	247/249 (99%)	1	between Bm10776 and Bm8585
6^¶^	250	161/161 (100%)	3	5s rRNA array
7^¶^	280	179/182 (98%)	3	5s rRNA array
8	420	321/321 (100%)	3	between Bm5804 and Bm17591

* These transposons inserted into the same intergenic region approximately 1.5 kbp apart.

^¶^ These transposons inserted into two different sites within the spacer of the 5s rRNA gene array. The exact insertion site was therefore impossible to determine.

**Fig 4:**
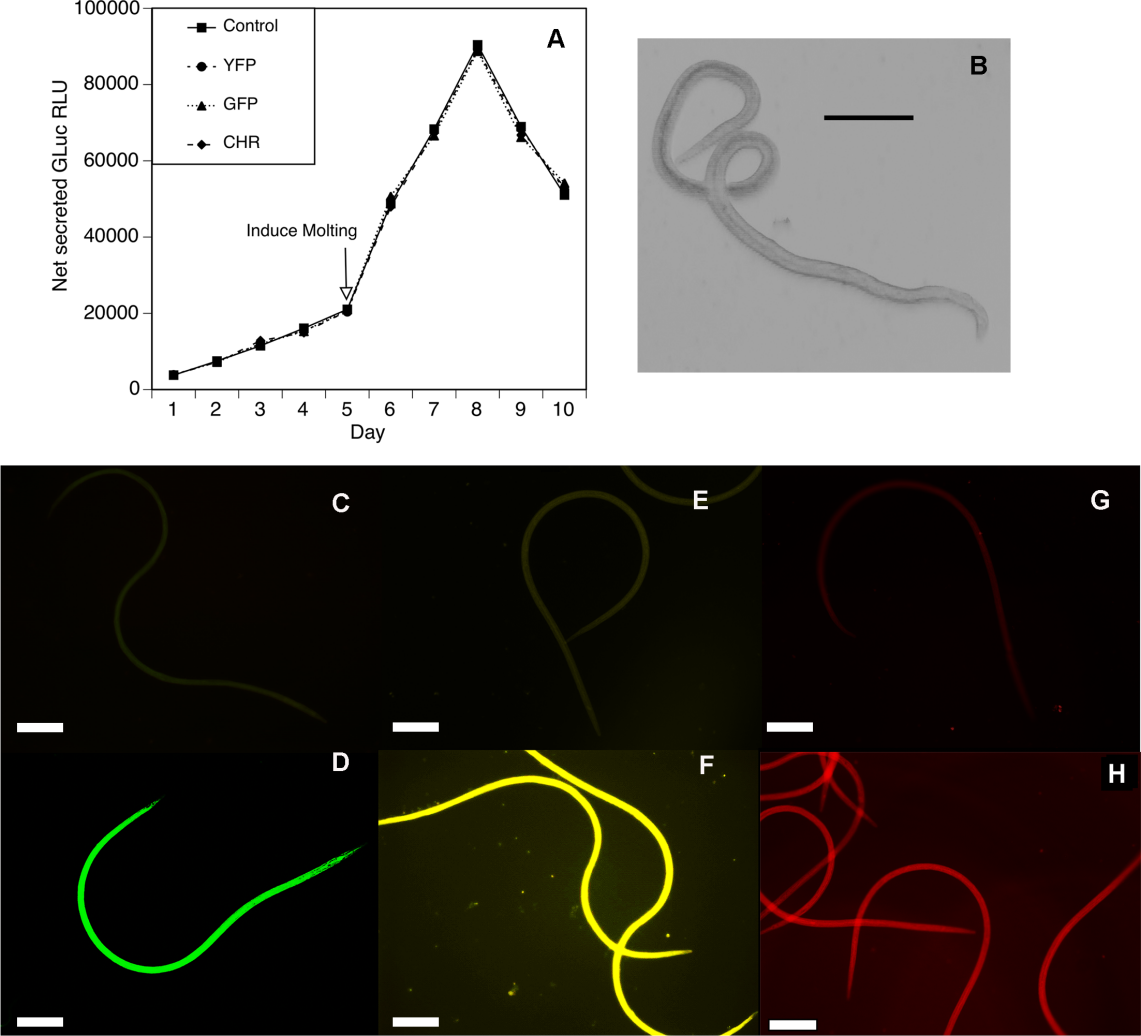
Secreted luciferase activity and fluorescent signal on L3 transfected with dual reporter plasmids. L3 were transfected with pBACII-BmGluc-MCS, pBACII-BmGluc-GFP, pBACII-BmGluc-YFP, or pBACII-BmGluc-CHR as described in the text. (A) Secreted luciferase activity in cultures of transfected larvae. The arrow indicates the day upon which molting was induced. Control = L3 transfected with pBACII-BmGluc-MCS, GFP = L3 transfected with pBACII-BmGluc-GFP, YFP = L3 transfected with pBACII-BmGluc-YFP and CHR = L3 transfected with pBACII-BmGluc-CHR. (B) Brightfield image of transfected parasites on day 8. (C) GFP channel florescence in the L3 transfected with pBACII-BmGluc-MCS. (D) GFP channel florescence in the L3 transfected with pBACII-BmGluc-GFP. (E) YFP channel florescence in the L3 transfected with pBACII-BmGluc-MCS. (F) YFP channel florescence in the L3 transfected with pBACII-BmGluc-YFP. (G) CHR channel florescence in the L3 transfected with pBACII-BmGluc-MCS. (H) CHR channel florescence in the L3 transfected with pBACII-BmGluc-CHR. In panels B-H bar = 300nm.

Vectors expressing fluorescent proteins have been used effectively to study protein spatial and temporal expression, trafficking and regulation in *Caenorhabditis elegans* [20–25] and in other nematode parasites [26, 27]. As a first step in developing constructs that could express fluorescent protein tags, the *piggyBac* construct containing the *GLuc* marker described above was modified to include a poly-linker located downstream of the 3’ end of the selectable marker cassette. We cloned the *B*. *malayi* 12 kDa small subunit ribosomal protein (Bm*rps12*) promoter and 3’ UTR into the 5' and 3' restriction sites of the polylinker and then inserted ORFs for green fluorescent protein (GFP), yellow fluorescent protein (YFP) and cherryRed (CHR) into a restriction site located between the promoter and 3’ UTR, creating three plasmids each containing the GLuc selectable marker and a different fluorescent protein ORF. We chose the *Bmrps12* promoter and 3’ UTR for these studies, as previous work demonstrated that it is a strong constitutive promoter [15]. The three constructs were then transfected into developing larvae exactly as described previously, along with the construct containing the Bm*rps12* promoter and 3' UTR but lacking the fluorescent protein ORFs as a negative control. The culture media was assayed for secreted Gluc activity (as a control for transfection efficiency) and the larvae examined for expression of the fluorescent proteins by fluorescent microscopy eight days after transfections began. Secreted GLuc activity was almost identical in all transfected parasites, demonstrating an equivalent lipofection efficiency with all constructs (Fig 4A). Parasites transfected with all three fluorescent tags exhibited signals of the appropriate color, while no signal was seen in parasites transfected with the construct lacking a fluorescent tag (Fig 4C–4H). All larvae from examined from the cultures lipofected with the constructs containing the fluorescent reporter constructs (n = 25 per lipofection) exhibited the same uniform pattern of fluorescence as shown in Fig 4, although the intensity of the signal varied slightly among individual larva (c.f. Fig 4H).

## Discussion

Taken together these studies represent a major advance in utilizing modern genetic tools to study human filarial parasites. First, we report that co-culture of *B*. *malayi* L3 with BESM cells resulted in parasites that were as developmentally competent as fresh L3 when introduced into naive animal hosts. Second, the co-cultured L3 were efficiently transfected by lipofection. Third, the *piggyBac* transposon system could be used to produce integrative transfectants when employed on the co-cultured L3. Fourth, we demonstrated that a secreted luciferase can be used as a selectable marker to identify individual transgenic parasites. Finally, we have developed a number of constructs that can be used to take advantage of these advances, including dual reporter vectors that will be useful in studies of transcriptional and post-transcriptional regulation of parasite genes, both at the temporal and spatial scales.

Similar to previous studies transfecting L3 not subject to co-culture [7], we observed an increase in reporter activity when the L3 were induced to molt in culture, suggesting transfection efficiency was increased in molting parasites. It is possible that this is due to the fact that cuticle maturation is a process that is not complete at the molt, but continues for some time thereafter [28]. This post-molting maturation process involves extracellular crosslinking of the cuticular collagens that increase the strength and rigidity of the cuticle. Mutation of enzymes involved in this process results in parasites whose cuticles exhibit a variety of morphological defects and which are more permeable to macromolecules than wild type animals [29]. The enhanced permeability of the immature cuticles in the freshly molted parasites may be why molting seems enhance lipofection efficiency.

The fluorescent reporter constructs tested here all gave strong uniform signals in the eight-day larvae. This is perhaps not surprising for two reasons. First, the Bm*rps12* promoter used to drive the expression on the fluorescent reporter genes is derived from a ribosomal protein gene and thus would be expected to be strongly expressed in all cells and in all life cycle stages. Second, the hypodermis, the tissue responsible for the synthesis of the cuticle, is the structure that lies directly below the cuticle. In nematodes, the hypodermis consists of a large multi-nucleated syncytium, which is effectively a single large multi-nucleated cell [30, 31]. Successful transfection of the hypodermal nucleii would result in expression and diffusion of any reporter throughout this syncytium, resulting in the uniform pattern we observed.

In the studies described above, we demonstrated that the secreted Gaussia luciferase could be used as a selectable marker to identify individual F1 generation microfilaria. Furthermore, recovery of adult parasites derived from transfected larvae carrying a construct expressing the secreted GLuc was similar to recovery of adult parasites transfected with fresh untransfected larvae. Thus, it seems that the GLuc marker did not have a significant detrimental effect on the development of the transfected larvae. However, we have not yet attempted to infect mosquitoes with the transgenic microfilariae and produce infective larvae. It is possible that GLuc secretion may have a detrimental effect on parasite development in the insect vector and it may therefore not be an ideal selectable marker. It this is the case, it should be possible to use a fluorescent reporter as a selectable marker, either by manually selecting transgenic fluorescent parasites or by collection of fluorescent parasites using automated methods [32].

The constructs and methods described here should be immediately applicable to many studies that involve "knocking in" genes in the human filaria. For example, it is easy to envision the application of this technology to the study of promoter elements involved in tissue and stage-specific gene expression. The methods may also be immediately applied to the verification of potential alleles thought to be involved in the development of drug resistance, at least in cases where resistance is thought to be a dominant phenotype. Such experiments could be used to definitively identify resistance loci, which could in turn be used to develop diagnostic tests to rapidly detect and counteract the development of resistance to the limited number of drugs that are currently used by the international efforts attempting to eliminate these diseases.

In all the individual microfilaria examined in this study, a single distinct band was produced in the TAIL PCR assays used to determine the integration sites of the *piggyBac* reporter. This suggests that a single integration event had occurred for each individual microfilaria. However, it is possible that multiple integration events had occurred and the TAIL PCR recovered only the most efficiently amplified junction sequence. Definitively demonstrating that a single integration event had occurred would require carrying out whole genome sequencing on individual microfilaria, as the amount of genomic DNA present in an individual microfilaria is not sufficient for conventional Southern Blot analysis.

While the *piggyBac* system is useful for knocking in genes (and for the development of random knockout libraries [13]), it suffers from some disadvantages. First, the semi-random nature of the insertion points means that the transposon can enter and disrupt genes, which could be detrimental to the long-term viability and fecundity of a transgenic parasite line. Second, the random nature of the insertion process can result in position effects that can affect transgene expression, a problem for which it can be difficult to control. Third, the *piggyBac* system cannot precisely target specific sequences in the genome, making precise generation of knockout or replacement mutations using this system impossible. Clustered Regularly Interspaced Short Palindromic Repeats (CRISPR) based technology [33], which permits precise targeting (and editing) of particular sequences in the genome is useful for these sorts of applications. CRISPR technology has recently been successfully applied to *Strongyloides stercoralis* [34] and it is likely that the methods and constructs described here can be adapted for use with CRISPR technologies. Efforts to develop a CRISPR based system based upon the advances described here are currently underway.

While the methods and constructs reported here represent a major step in the decades long effort to develop reverse genetics in the human filaria, more work is needed before reverse genetics can be easily and efficiently applied to these parasites. First, it will be important in the long term to be able to develop parasite lines which are homozygous for the transgene, especially in situations where a recessive phenotype is produced. This will require developing methods to non-invasively sex and genotype parasites and to carry out defined mating experiments with genotyped individuals to establish homozygous parasite lines. Second, since these are obligate parasites, maintaining a transgenic parasite line will require continuous passage through both the mammalian and mosquito hosts, something that will be expensive to do for more than a few lines. Developing an efficient method to cryopreserve transgenic *B*. *malayi* will therefore be a priority.

## Supporting information

S1 Fig Summary of construction of pBACII-BmGluc-MCS. (TIF)

S2 Fig Summary of construction of pBmCDTH. (TIF)

S3 Fig Summary of construction of pBACII-BmGluc-GFP, pBACII-BmGluc-YFP and pBACII-BmGluc-CHR. (TIF)
