# Supplementary figures and images for: Development of a toolkit for piggyBac-mediated integrative transfection of the human filarial parasite Brugia malayi
Source: PLoS Negl Trop Dis. 2018 May 21;12(5):e0006509. doi: 10.1371/journal.pntd.0006509 (PMC5983866; doi:10.1371/journal.pntd.0006509)

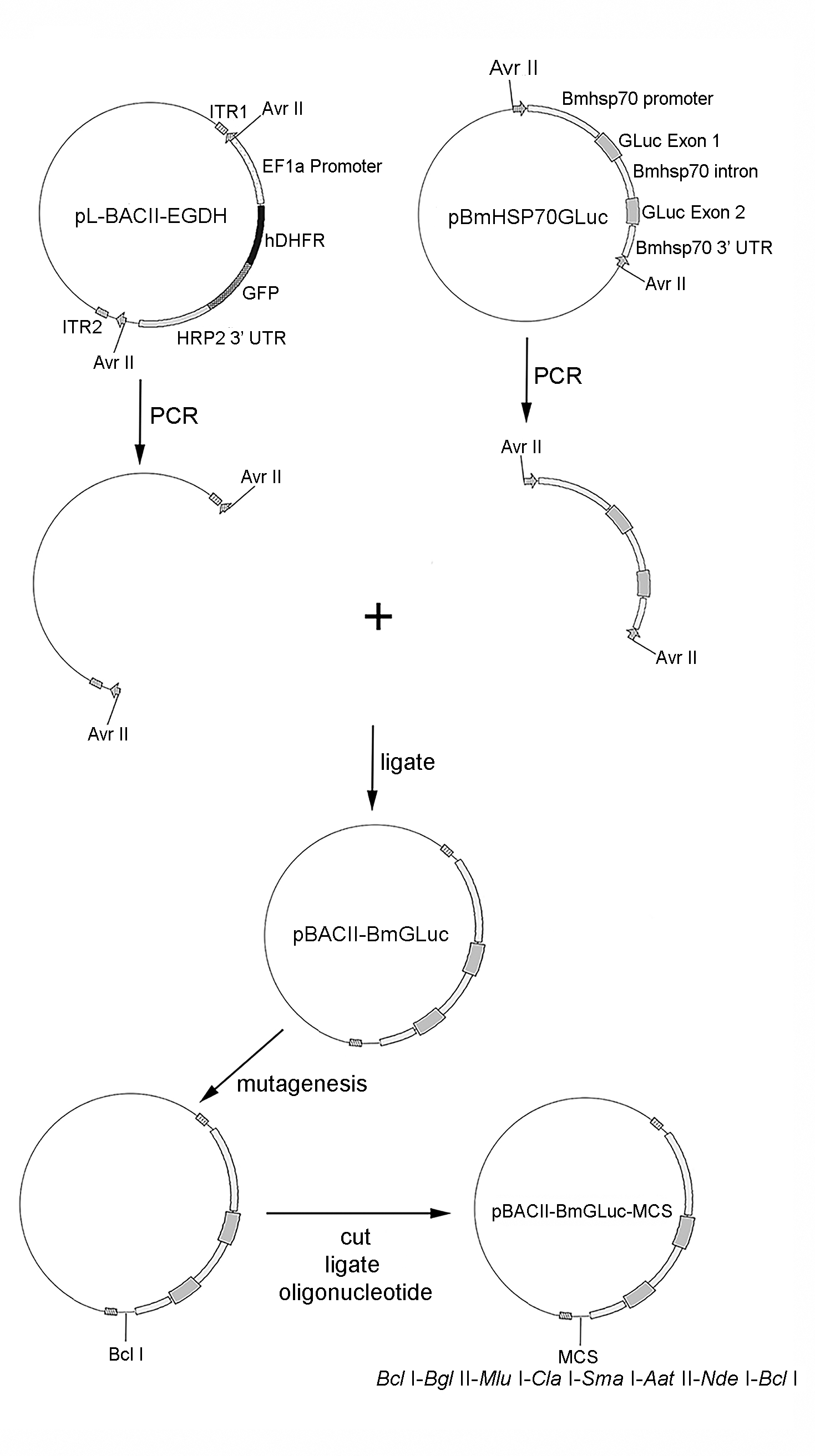

Supplement: S1 Fig — (TIF) [file pntd.0006509.s001.tif]

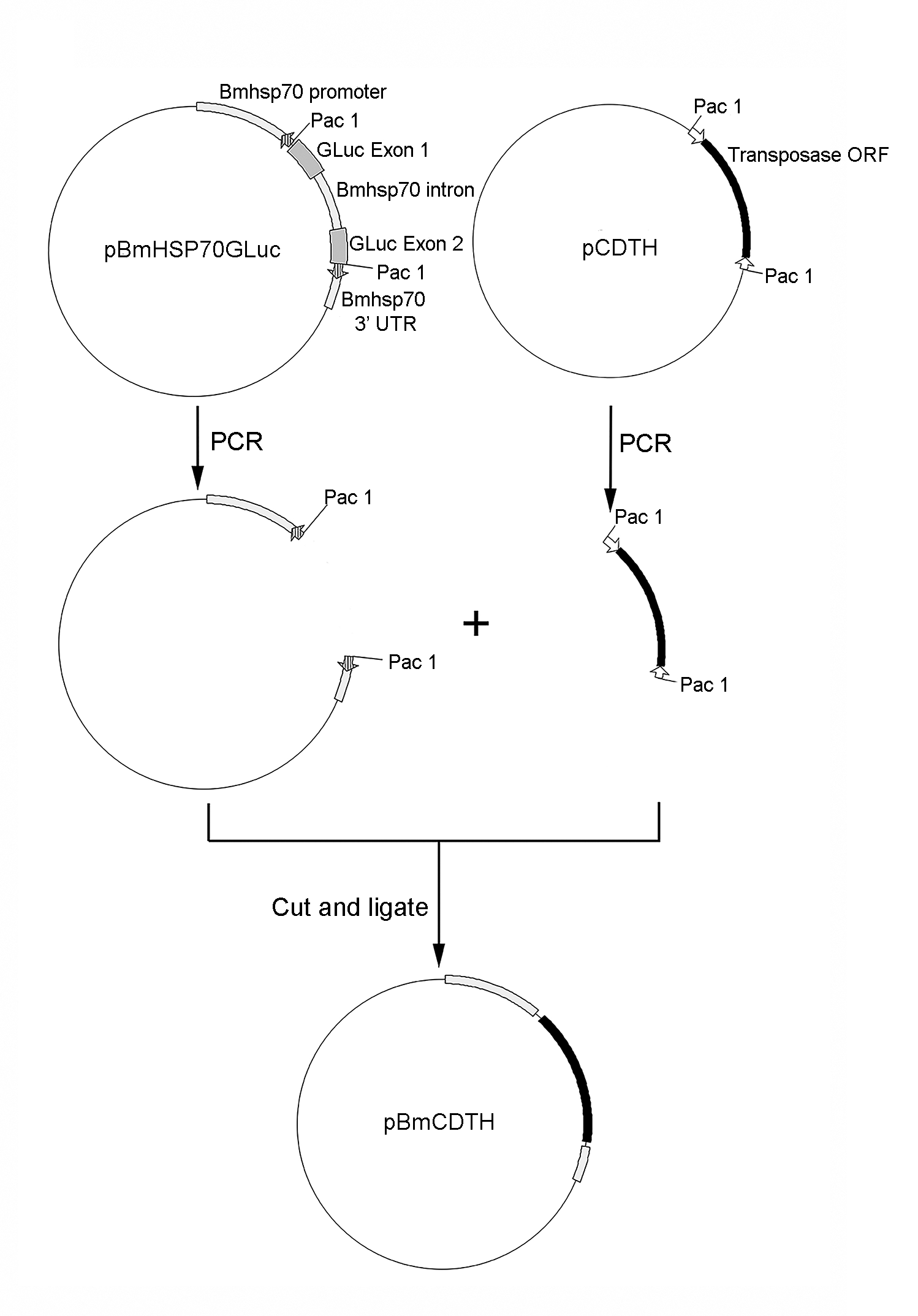

Supplement: S2 Fig — (TIF) [file pntd.0006509.s002.tif]

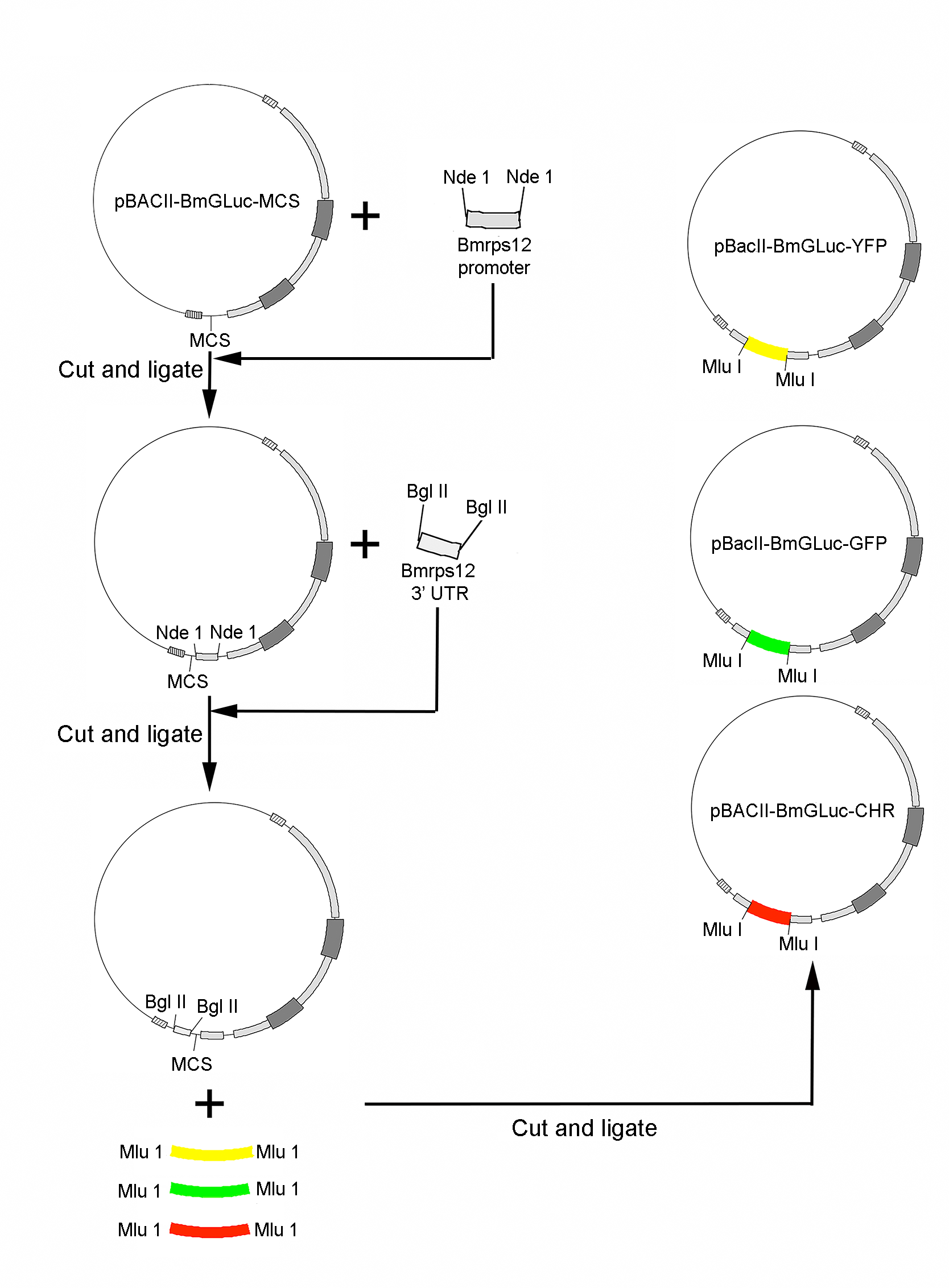

Supplement: S3 Fig — (TIF) [file pntd.0006509.s003.tif]
